# A Comparison between Droplet Digital and Quantitative PCR in the Analysis of Bacterial 16S Load in Lung Tissue Samples from Control and COPD GOLD 2

**DOI:** 10.1371/journal.pone.0110351

**Published:** 2014-10-16

**Authors:** Marc A. Sze, Meysam Abbasi, James C. Hogg, Don D. Sin

**Affiliations:** 1 Centre for Heart Lung Innovation, St. Paul's Hospital, Departments of Medicine and Pathology and Laboratory Medicine, University of British Columbia, Vancouver, BC, Canada; 2 BioRad Laboratories, Mississauga, ON, Canada; University of Padova, Medical School, Italy

## Abstract

**Background:**

Low biomass in the bacterial lung tissue microbiome utilizes quantitative PCR (qPCR) 16S bacterial assays at their limit of detection. New technology like droplet digital PCR (ddPCR) could allow for higher sensitivity and accuracy of quantification. These attributes are needed if specific bacteria within the bacterial lung tissue microbiome are to be evaluated as potential contributors to diseases such as chronic obstructive pulmonary disease (COPD). We hypothesize that ddPCR is better at quantifying the total bacterial load in lung tissue versus qPCR.

**Methods:**

Control (n = 16) and COPD GOLD 2 (n = 16) tissue samples were obtained from patients who underwent lung resection surgery, were cut on a cryotome, and sections were assigned for use in quantitative histology or for DNA extraction. qPCR and ddPCR were performed on these samples using primers spanning the V2 region on the 16S rRNA gene along with negative controls. Total 16S counts were compared between the two methods. Both methods were assessed for correlations with quantitative histology measurements of the tissue.

**Results:**

There was no difference in the average total 16S counts (P>0.05) between the two methods. However, the negative controls contained significantly lower counts in the ddPCR (0.55 ± 0.28 16S/uL) than in the qPCR assay (1.00 ± 0.70 16S copies) (P <0.05). The coefficient of variation was significantly lower for the ddPCR assay (0.18 ± 0.14) versus the qPCR assay (0.62 ± 0.29) (P<0.05).

**Conclusion:**

Overall the ddPCR 16S assay performed better by reducing the background noise in 16S of the negative controls compared with 16S qPCR assay.

## Introduction

Recently, we reported that lung tissue samples of smokers, non-smokers and those with, chronic obstructive pulmonary disease (COPD), and cystic fibrosis (CF) showed increased bacterial population compared with controls [1]. We used qPCR quantitation of 16S rRNA to detect levels of bacterial microbiome in these samples. For absolute quantitation of 16S rRNA, serial dilution of *Escherichia coli* (E-coli) DNA was required for generation of a standard curve on every plate. This process can be time consuming and costly, and limits sample throughput. Moreover, one needs to ensure that the standard curve is optimized and contains an effective dynamic range for accurate quantitation of target genes in desired samples [2]. Often, results could be misleading as the reaction efficiency of the standard samples may vary from the reaction efficiency of test samples due to differences in sample content and presence of inhibitors [3], [4]. The requirement for a large number of technical replicates when assessing low abundance genes is another major hurdle associated with this technique, which could be problematic when amount of sample is limited [5]. The concentration of 16S rRNA in lung tissue samples is extremely low (1–10 copies/µL), and very close to the lower detection limit of qPCR. Precise and accurate measurement of the low copies of 16S rRNA in lung tissues is essential to differentiate between negative controls, smokers, non-smokers, COPD, and CF samples. For this purpose, a more precise method is required for 16S rRNA quantification.

Droplet digital PCR (ddPCR) allows for absolute quantitation of nucleic acids without the requirement for standard curves. The technique is based on partitioning of a single sample into 20,000 much smaller, segregated reaction vessels (known as droplets). A standard PCR reaction can then be employed to amplify the target(s) in each droplet which can be individually counted by the associated target dependant fluorescence signal as positive or negative. The simple readout of droplet partitions as a binary code of ones (positive) and zeroes (negative) represents the “digital” aspect of the technique and because the presence of a target in a given droplet is a random event, the associated data fits a Poisson distribution [6], [7]. This permits the direct and simple calculation of DNA copy numbers in a sample without the requirement of a standard curve. Since ddPCR is an end point PCR reaction, data are not affected by variations in reaction efficiency and as long as the amplified droplets display increased fluorescence intensity compared to the negative droplets, absolute copy number of target genes can be obtained with a high degree of confidence. Owing to the high precision and accuracy of this technique, the need for technical replicates is reduced [8], and the Poisson distribution provides 95% confidence intervals for measured copies from single wells which provides robust estimates of data dispersion obtained from technical replicates [9]. This can significantly increase sample throughput, save time, and effectively allow accurate quantitation of precious samples.

Sample partitioning in ddPCR also improves sensitivity when quantifying low concentration of target genes in a highly concentrated complex background [8], [10], [11]. When quantifying a low amount of 16S bacterial rRNA in DNA extracted from human lung tissue, the 16S primers have a difficult task of browsing through the large number of non-specific sequences contained in the complementary strand. This reduces sensitivity of the assay by introducing noise in target amplification. By using ddPCR to partition sample into 20,000 droplets we are able to increase the signal to background ratio by a factor of 20,000 and the primers and probes are able to locate the target sequence from a far less concentrated background. Using this technique, we aim to increase accuracy and sensitivity in detecting total bacteria within the lung of smokers, non-smokers, and COPD patients.

## Methods

### Tissue Samples

Lung tissue was obtained from the tissue registry at St. Paul's Hospital. Ethics approval was specifically obtained for this study from the University of British Columbia - Providence Health Care (UBC-PHC) Research ethics board. Informed consent was obtained, through a written consent form, and approved by the UBC-PHC Research ethics board for patients who underwent lung resection therapy for various pulmonary conditions, such as lung cancer, for collection and use in this study. For this study, we used lung tissue from the tumor-free part of the resected lung segment. Samples were obtained from 16 control (FEV1/FVC>0.7) and 16 patients with moderate COPD GOLD 2 (Global initiative for chronic Obstructive Lung Disease) (FEV1/FVC <0.7, and 50% <FEV1 <80%) were used. Resected lung tissues were inflated with cryomatrix (OCT) and then frozen in liquid nitrogen. From this, 2cm thick contiguous transverse slices were made and tissue samples were taken from one of these slices. Frozen sections were obtained by cutting the tissue sample on a cryotome with some sections assigned for DNA extraction and others used for quantitative histology [12].

### Experimental Protocol

DNA from all samples was extracted using a Qiagen DNeasy Extraction kit according to the manufacturer's instructions and the concentration was assessed using Nanodrop. qPCR (Applied Biosystems ViiA7) was performed on these samples using a previously published 16S rRNA assay [1] that utilized a standard curve of a serial dilution of *Escherichia coli* (cycling conditions were 1 cycle at 95°C for 15 minutes, 40 cycles at 95°C for 15 seconds and 60°C for 1 minute, followed by a standard denaturation curve protocol). The assay was a SYBR green qPCR assay and three replicates were used per sample. Data were collected using the ABI ViiA7 RUO software program. The same assay was adapted to ddPCR (Bio-Rad QX200) and the experiments were performed using the following protocol: 1 cycle at 95°C for 5 minutes, 40 cycles at 95°C for 15 seconds and 60°C for 1 minute, 1 cycle at 4°C for 5 minutes, and 1 cycle at 90°C for 5 minutes all at a ramp rate of 2°C/second. Bio-Rad's T100 thermal cycler was used for the PCR step. No standard curve was required for the ddPCR and the droplets were quantified using the Bio-Rad Quantisoft software. A total of two replicates were used per sample. A threshold cutoff of 20000 was chosen based on preliminary experiments, which accurately separated positive from negative droplets. For both protocols, negative controls that comprised of DNase and RNase free water were used and were run alongside the samples.

### Quantitative Histology

Sections were stained with Movat pentachrome stain and Hematoxylin and Eosin (H&E) to obtain the mean linear intercept (Lm) which is a marker of emphysematous destruction of airspaces [13], [14]. The arithmetic mean of the Lm obtained from the Movat pentachrome and H&E stained sections were used as the analytic value of Lm for each tissue sample. Immunohistochemical staining for both the small airway and alveolar volume fraction (Vv) of CD4 T-cells, CD8 T-cells, B-cells, macrophages, and neutrophils (PMN) were obtained by using a grid based point counting method to obtain a positive cell:tissue ratio for each cell type [15].

### Data Analysis

Analysis involved testing whether ddPCR and qPCR protocols could differentiate 16S in tissue samples from those of the negative controls. Direct comparison of the total 16S obtained with both methods was made to detect differences between tissue samples and negative controls. The coefficient of variation between ddPCR and qPCR methods was then compared. Finally, the data generated from both techniques were compared with important histological measures of COPD to determine the relationship of 16S findings from ddPCR and qPCR with parameters of COPD. Grouped analysis used Kruskall-wallis ANOVA analysis with Tukey's post hoc testing. Standard t-tests were used in non-grouped analysis. A P-value <0.05 was considered statistically significant and all analysis was performed using Prism v. 5 (GraphPad Software Inc. La Jolla California).

## Results

### 16S Detection with qPCR or ddPCR

**Figure 1:**
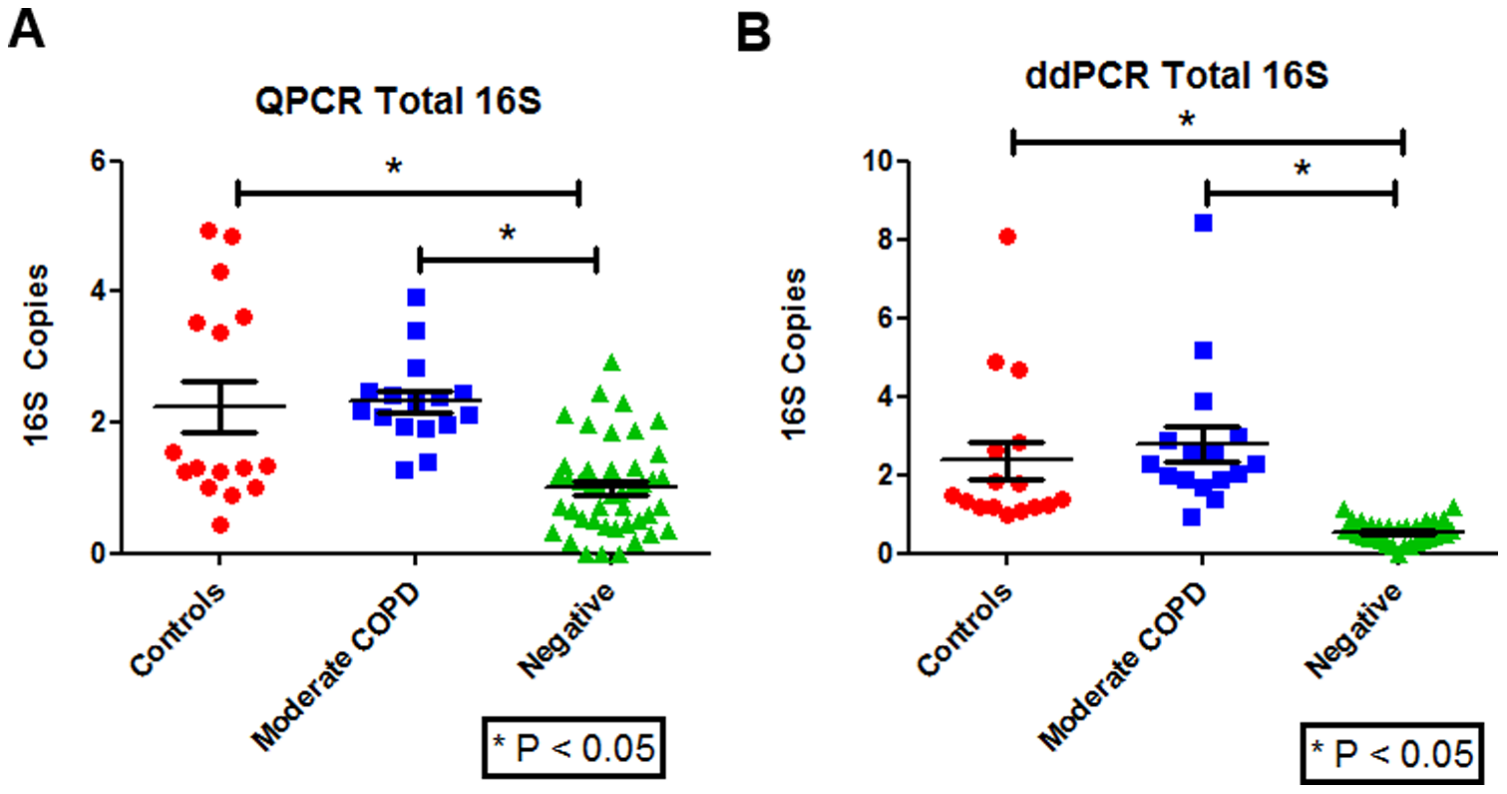
Overall total bacterial 16S load measured in lung tissue using qPCR or ddPCR. A) qPCR results in controls, moderate COPD, and negative non-template controls (water negative). The total bacterial 16S load was significantly lower in negative controls compared with moderate COPD and control lung tissue. B) ddPCR results in controls, moderate COPD, and negative non-template controls. The total bacterial 16S load was significantly lower in negative controls compared with moderate COPD and control lung tissue.

Both qPCR and ddPCR were assessed for their ability to detect 16S and whether the samples were above the negative non-template control samples. Figure 1A shows that the qPCR assay was able to detect the bacterial 16S rRNA gene and that both controls and moderate COPD samples were significantly higher than that of the negative controls (P <0.05). Figure 1B shows that the ddPCR could also detect the bacterial 16S rRNA gene and that both controls and moderate COPD samples were significantly higher than the negative controls (P <0.05). Both methods showed no significant difference in total 16S bacterial load between control and moderate COPD (P> 0.05).

### Comparison of the qPCR to ddPCR 16S rRNA Assay

**Figure 2:**
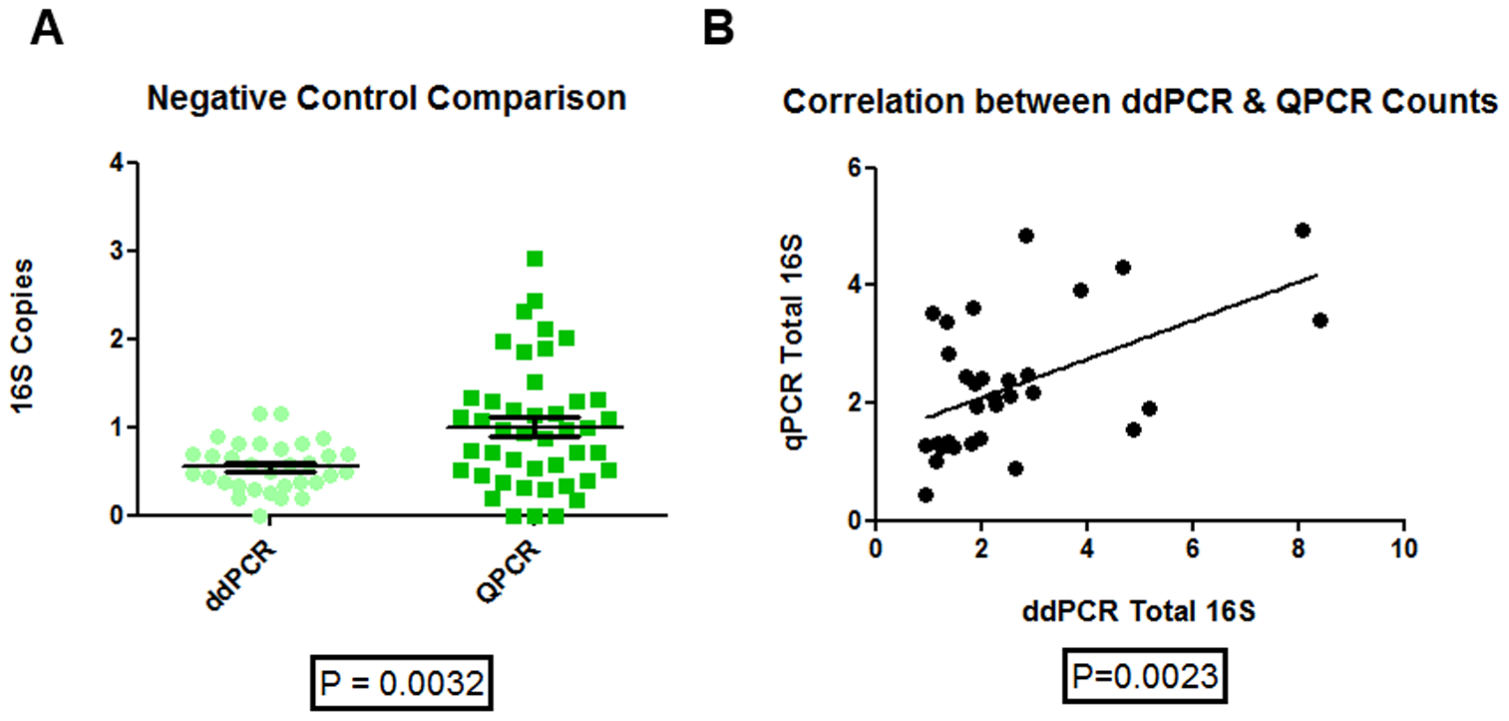
Negative control and direct 16S ddPCR versus qPCR comparisons. A) Direct comparison of the negative control for total bacterial 16S count between ddPCR and qPCR. There was a significant difference between the ddPCR and qPCR negative controls with ddPCR being on average lower. B) The linear correlation between the qPCR and ddPCR results were significant (y  =  0.33× + 1.44, R^2^ of 0.27).

**Figure 3:**
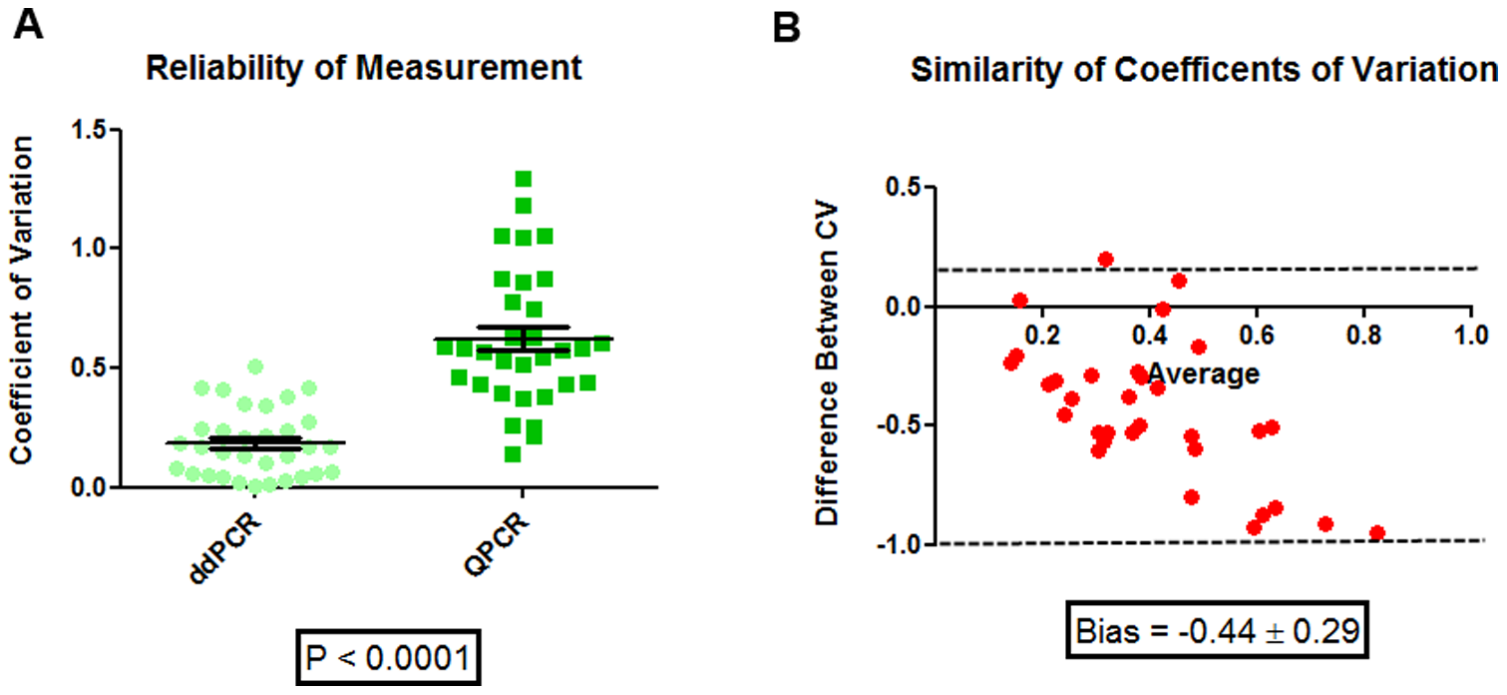
Comparison of the Coefficients of Variation (CV) between QPCR and ddPCR. A) Direct comparison of the ddPCR CV against the QPCR CV. The ddPCR CV was significantly lower than those obtained using qPCR. B) A Bland-Altman plot of the ddPCR CV against the qPCR CV. On average there was a much larger CV for the qPCR than the ddPCR for each individual sample of 0.44 ± 0.29.

The ddPCR negative controls had a much smaller standard deviation versus the qPCR negative controls (0.28 versus 0.70). Both the qPCR and ddPCR detected a similar bacterial load for the control and moderate COPD groups. For the moderate COPD group, qPCR values were 2.32 ± 0.67 16S copies (mean ± SD) and ddPCR values were 2.80 ± 1.80 16S/uL and for the control group the qPCR and ddPCR values were 2.25 ± 1.55 16S copies and 2.36 ± 1.95 16S/uL (mean ± SD) respectively. There was a significant decrease in the negative control 16S bacterial load using the ddPCR technique compared with qPCR (P <0.0032). The ddPCR had a value of 0.55 ± 0.28 16S/uL and the qPCR having a value of 1.00 ± 0.70 16S copies [Figure 2A]. There was a significant positive relationship between the qPCR and ddPCR 16S counts with an R^2^ value of 0.27 [Figure 2B]; the line of best fit was y  =  0.33× + 1.44. Further, the ddPCR coefficients of variation (CV) were significantly lower than those obtained by the qPCR assay (P-value <0.0001) [Figure 3A]. The average CV for the ddPCR was 0.18 ± 0.14 while for the same samples the CV for the qPCR was 0.62 ± 0.29. Using a Bland-Altman plot to further analyze the CV data and using the ddPCR as the reference against the qPCR the bias was found to be −0.44 ± 0.29. This means that on average for any given sample the qPCR CV will be 0.44 ± 0.29 higher than the ddPCR CV [Figure 3B].

### Comparison of the qPCR to ddPCR 16S rRNA Assay and correlations to important tissue measurements of COPD

**Figure 4:**
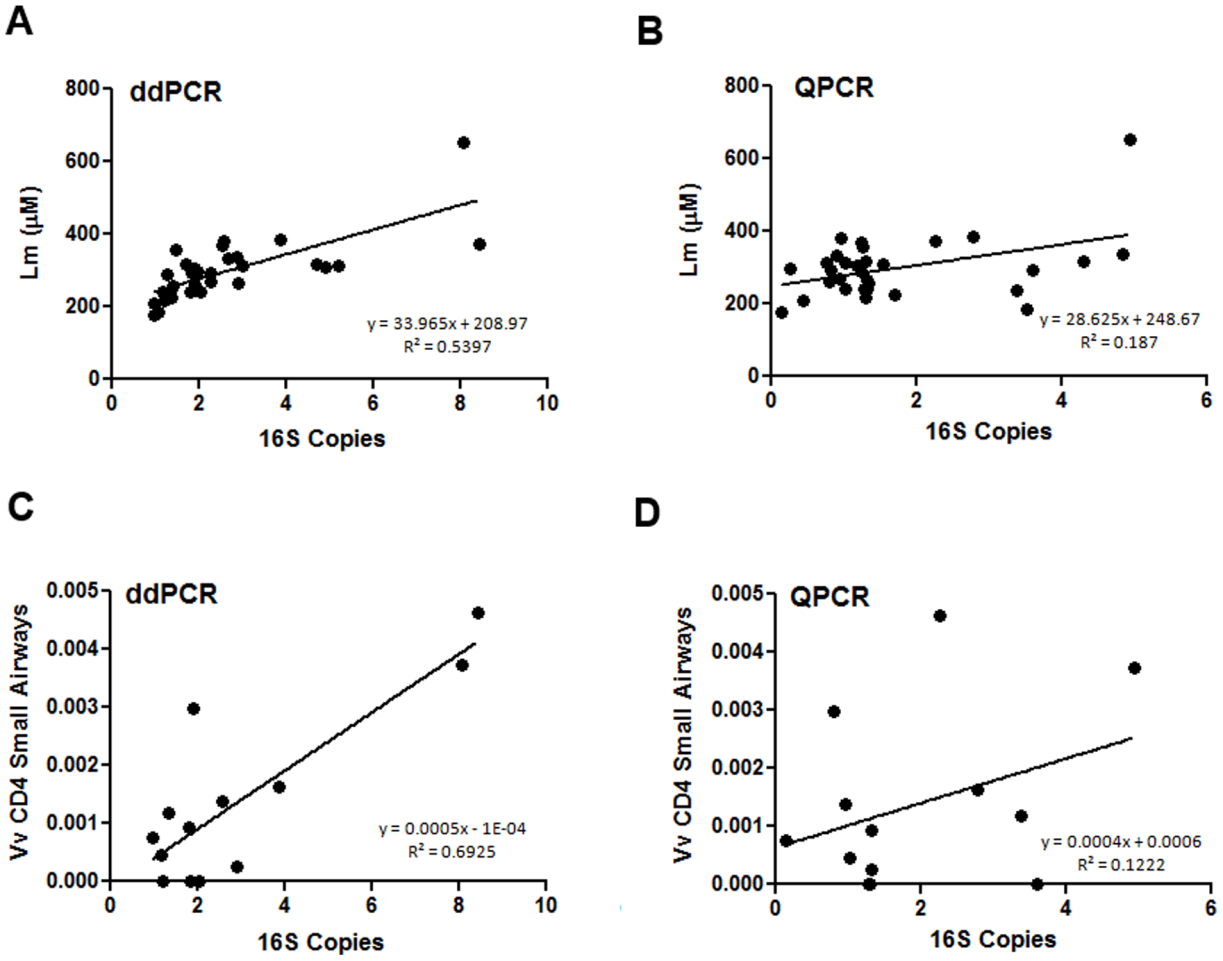
The relationship between quantitative histology parameters and ddPCR or QPCR measurements. A) ddPCR 16S bacterial loads versus the mean linear intercept (Lm). B) qPCR 16S bacterial loads versus the Lm. C) ddPCR 16S bacterial loads versus the small airway CD4 T-cell volume fraction (Vv). D) qPCR 16S bacterial loads versus the small airway CD4 T-cell Vv.

Using quantitative histology [15], we examined the relationship between ddPCR values and qPCR values and parameters of tissue remodeling and lung inflammation, which are salient histologic features of COPD. Both methods generally were similar when there was no significant correlation between the 16S counts and histologic measurements for tissue remodeling or inflammation (P>0.05). However, correlations with CD4 that was not previously significant using qPCR became significant when we used ddPCR [Figure 4C & D]. When there were significant correlations (P<0.05) ddPCR data was more tightly associated with the histologic measures of tissue remodeling compared with qPCR [Figure 4A & B]. Overall ddPCR demonstrated a larger slope than qPCR and also tended to have a greater dynamic range [Figure 4]. Figure 4A and 4B show the improved correlation between emphysematous tissue destruction (Lm) and total 16S bacterial counts with ddPCR (P-value <0.0001, R^2^  =  0.54) versus qPCR (P-value  =  0.015, R^2^  =  0.19). Similarly, figure 4C and 4D show the improved correlation between infiltration CD4 T-cells into the alveolar tissue and total 16S bacterial counts with ddPCR (P-value  =  0.0004, R^2^  =  0.69) versus qPCR (P- value  =  0.242, R^2^  =  0.12).

## Discussion

The first bacterial microbiome papers of the lungs were generated from materials obtained in bronchoalveolar lavage (BAL) and bronchial brushings [16], [17]. The total bacterial counts ranged from 10^3^ to 10^5^ total 16S within the lung [16]–[19]. However, when similar assays were performed in resected lung tissue, these counts dropped to ranges between 1 and 10^2^ total 16S per lung [1]. The lower range of bacterial 16S impinges on the lower limit of detection for traditional qPCR assays and as such cannot be accurately quantified using this technique. In this study, we determined whether ddPCR significantly improves detection of bacterial load compared with traditional techniques of quantification. Compared with traditional qPCR, ddPCR has lower detection limits and a larger dynamic range of detection. Consistent with these properties, we found that the ddPCR assay reduced CV and thus the noise to signal ratio of bacterial detection, enabling robust quantification [2]. This is important because although there were no significant difference in total bacterial count in control and moderate COPD tissue samples [Figure 1], the ddPCR technique improved the tightness and dynamic range of the relationship between total bacterial count and important parameters of COPD such as Lm and CD4 counts in the small airways [Figure 4].

To date, most papers have not found a significant difference between the total bacterial load and COPD [1], [16], [20]. However, there may be subtle but important differences in diversity of the bacterial microbiome between normal lungs and COPD lungs that might affect disease pathogenesis and progression [19], [21]. Our data suggest that using more sensitive PCR technology (ddPCR), we may gain important insights into potential disease mechanisms that may have been elusive using the traditional qPCR approach. This approach would also be a way of validating or investigating specific bacterial species identified from unbiased sequencing and their potential role in COPD disease pathogenesis. ddPCR may be the preferred method given that many important OTUs (Operational Taxonomic Units) identified in these previous studies were found in very low relative abundance [1], [17], [21]. The traditional qPCR technique may not have the ability to differentiate the samples owing to its relatively high detection limits.

Another potential application of this technology may be in evaluating a select number of bacterial species in longitudinal studies in lieu of full Roche 454 pyrotag or MiSeq Illumina sequencing, which are expensive. ddPCR may provide the needed sensitivity to follow specific low abundance bacterial species over time. The major advantage of ddPCR is in samples that contain relatively low abundance of bacterial load [Figure 3A]. Moving the lung microbiome field beyond the cross-sectional experimental design (to longitudinal studies) has been one of the major limitations in discovering and confirming the important bacterial genera and species involved in the pathogenesis of the disease [22]–[24] and may provide the crucial technology needed to assess specific bacteria within the tertiary lymphoid follicles seen in very severe COPD.

This improvement may not be limited to the bacterial microbiome, ddPCR may be useful in detecting low copies of specific virus. ddPCR may also be able to analyze differences in bacterial strains and help to investigate the emergence of new strains [25], [26] or and how they interact with the microbiome to help drive COPD progression. Overall this promising technology provides a measurable improvement over the traditional qPCR bacterial 16S assays used in assessing the bacterial load.

There were some limitations to the present study and ddPCR. We used a SYBR-green based assay rather than TaqMan-probe based methods for bacterial load quantification, which is thought to be more sensitive and less variable than SYBR-green based techniques [27]. However, with strict standardization and optimization of procedures (as we did for the present study), these advantages of TaqMan-probe based assays over SYBR-green based methods largely disappear [28]. Therefore, it is likely that ddPCR would be superior to TaqMan-based PCR with higher precision and faster throughput. A head-to-head comparison between these two methods would be needed to validate this hypothesis. A limitation associated with ddPCR is that it does not work well with high abundance samples and specifically for concentrations higher than ∼ 10^5^ target copies [7]. This is due to the partitioning aspect of the technology, number of droplets generated, and the Poisson equation used to accurately measure the number of DNA copies in the samples [7]. Along these lines the samples need to be diluted below 10^5^ copies of the gene if ddPCR is to be used for target quantitation. Otherwise qPCR can be applied for measuring samples with high abundance. Additionally, using ddPCR, the sample processing time increases by approximately 45 minutes and depending on the number of samples the droplet read time adds 1–2 hours to the overall process. However, the increased time required to complete the assay may be an appropriate trade-off in low abundance samples because ddPCR is superior to the qPCR assay by allowing for extremely accurate quantification while reducing the overall 16S bacterial reads detected in the negative control background samples. ddPCR is a promising new technology that can potentially greatly advance the lung microbiome field by helping to move the field from hypothesis generating to hypothesis testing.
